# Traditional serrated adenoma of the sigmoid colon with osseous metaplasia: a case report

**DOI:** 10.1186/1752-1947-6-133

**Published:** 2012-05-23

**Authors:** Nelson F Montalvo, José N Beltrán, Ligia A Redrobán

**Affiliations:** 1 Servicio de Patología, Hospital Metropolitano, Av. Mariana de Jesús s/n y Nicolás Arteta, Quito, Ecuador; 2 Servicio de Patología, Hospital del IESS, Ibarra, Ecuador

## Abstract

**Introduction:**

Osseous metaplasia in the gastrointestinal tract is a rare phenomenon.

**Case presentation:**

We present the case of a 62-year-old Hispanic man with two colonic polypoid lesions, one of which, upon resection and histopathological examination, was found to be a traditional serrated adenoma with a focus of stromal osseous metaplasia.

**Conclusions:**

Our patient’s case is the third report of stromal osseous metaplasia in a traditional serrated adenoma of the sigmoid colon.

## Introduction

Epithelial metaplasia is a common occurrence in the gastrointestinal tract (GIT), especially in the upper GIT (excluding the liver and pancreas). However, mesenchymal metaplasia, particularly osseous metaplasia (OM), is a rare and incidental finding in the GIT and is composed of histologically benign bone tissue [1-3]. Fewer than 100 cases have been described in the literature. Gruber, in 1913, was the first to describe a case of osseous metaplasia in a gastric adenocarcinoma [4].

In 1939, Dukes described four cases of OM in colonic adenocarcinomas; since then, it has been described in both benign and malignant GIT lesions. The rectal region is particularly affected (adenocarcinoma), although the spectrum of presentation includes Barrett’s esophagus; tubular, tubulovillous and serrated adenomas; juvenile and hyperplastic polyps; and appendicular tumors (Table 1). A number of histopathological characteristics have been associated with OM development: mucin extravasation, chronic and active inflammation and/or ulceration, necrotic tissue, and stromal fibroblastic proliferation [5-9].

**Table 1:** Osseous metaplasia in gastrointestinal tract lesions

**Location**	**Histopathological diagnoses**	**References**
Esophagus	Barrett’s esophagus	[1,4]
Stomach	Hyperplastic polyp, carcinoid tumor, adenocarcinoma	[2-5]
Small intestine	Peutz-Jeghers syndrome, adenocarcinoma (peri-ampullary region)	[3,4,6,10]
Cecal appendix	Mucocele, mucinous cystadenocarcinoma, adenocarcinoma	[3-5]
Ascending colon	Metastatic signet-ring cell, adenocarcinoma	[4,5,7]
Transverse colon	Hyperplastic polyp, tubular adenoma, adenocarcinoma	[3,4,8,10-12]
Descending colon	Tubular adenoma, tubulovillous adenoma	[3,4,8,10,13]
Unspecified colon	Adenocarcinoma	[3,4,7,9,11,14]
Rectum	Proctocolitis, solitary rectal ulcer syndrome, adenoacanthoma, juvenile polyp, traditional serrated adenoma*, tubular adenoma, tubulovillous adenoma, adenocarcinoma with squamous metaplasia, adenocarcinoma arising in a traditional serrated adenoma	[1-5,8,9,11,12,14-16]

*Third case reported in the literature.

## Case presentation

A 62-year-old Hispanic man with no prior history of gastrointestinal problems presented for a routine endoscopic examination. He had no family history of colorectal cancer or polyps. He had a polyp 30 cm from the anal margin that was removed through open polypectomy and rectoscopy.

Macroscopically, the specimen was a 5 × 3.5 × 2 cm sessile polyp with a reddish, villous surface and a 4.5 × 2 cm implantation base. The tissue was fixed in 10% formol, embedded in paraffin, cut at 4 μm, and subsequently stained with hematoxylin and eosin.

**Figure 1:**
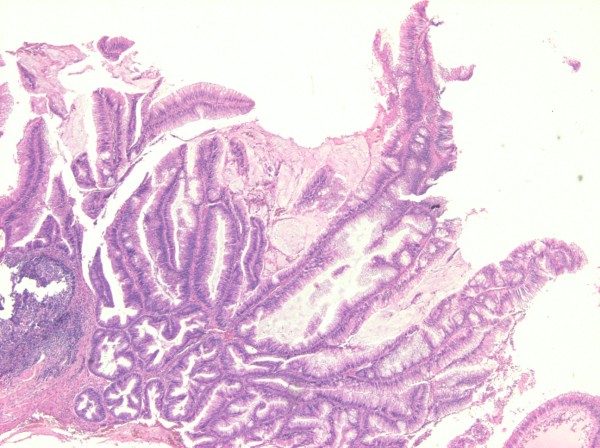
Traditional serrated adenoma of colonic mucosa (sigmoid) (hematoxylin and eosin staining, 2×).

**Figure 2:**
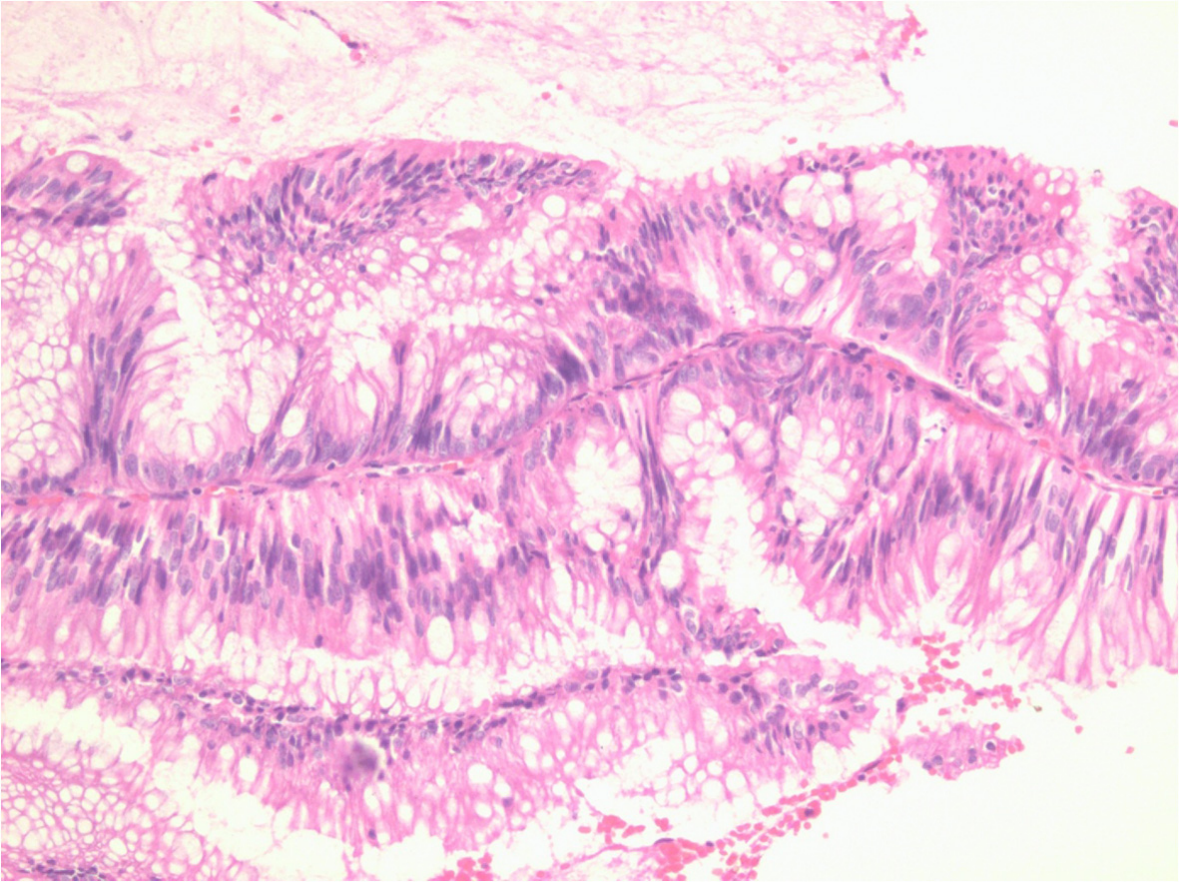
Cells lining the villi are eosinophilic with small ectopic crypts (hematoxylin and eosin staining, 10×).

**Figure 3:**
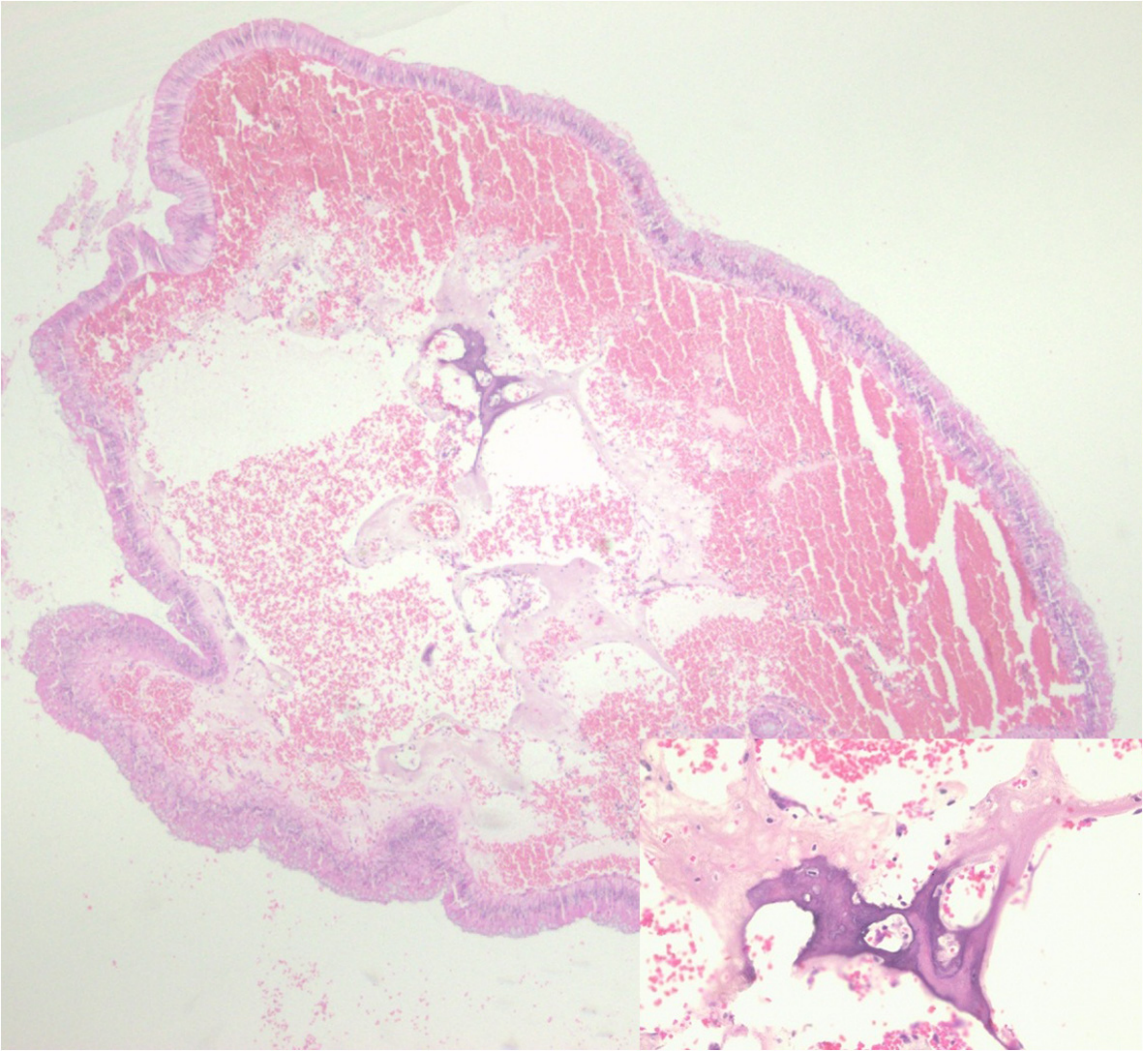
**Fibrovascular axis. **Inset: focus of osseous metaplasia (hematoxylin and eosin staining, 40×).

Microscopically, at low power the specimen was a traditional serrated adenoma (TSA) of colonic mucosa (sigmoid) with exuberant, villiform growth pattern and complex serration. High power examination disclosed eosinophilic pencillate cells, numerous ectopic crypts and low-grade epithelial dysplasia. (Figures 1 and 2) The fibrovascular axes showed numerous lymphangiectasias. In one of the axes a single small focus of stromal osseous metaplasia consisting of normal-looking osseous trabeculae with peripheral retraction and hemorrhage was detected. No characteristic was identified (chronic or active inflammation, ulceration, hyalinization or mucin extravasation) that would suggest a traumatic or ischemic process (Figure 3).

## Discussion

Mesenchymal metaplasia, particularly osseous metaplasia, is an unusual finding in GIT, composed of histologically benign bone tissue. This is an unexpected variation of the usual histological features encountered in colorectal TSA. In 1939, Dukes described four cases of OM in colonic adenocarcinoma; since then, it has been described in both benign and malignant GIT lesions, but the rectal region is particularly affected [1,3,8].

Despite the absence of a conclusive explanation of the induction mechanism of ossification, the hypothesis proposed by Rhone and Horowitz in 1976 postulated the metaplasia of a pluripotent cell into an osteoblast under the influence of factors generated by epithelial cells. One possible mechanism includes the production of bone morphogenetic proteins (BMP)-5 and BMP-6 by epithelial cells and BMP-2 and BMP-4 by adjacent fibroblasts [9-11,13,15,16].

An extensive review of the literature reveals that OM is mostly described in association with colorectal carcinomas and adenomas (both tubular and villous); however, no risk association has been determined for sex, age or polyp size [5,8,9,11]. This is the third reported case of OM in a traditional serrated adenoma. As in the first, there was no malignant transformation, in contrast to the second case reported, in which an adenocarcinoma had developed [12,14]. The site in all three cases was the left colon.

## Conclusions

According to our bibliographic review, our patient’s case is only the third report of osseous metaplasia in the stroma of a traditional serrated adenoma located in the sigmoid colon. No characteristic has been identified that would suggest a traumatic or ischemic process, nor any foci of calcification, either proximal or distal.

## Consent

Written informed consent was obtained from the patient for publication of this case report and accompanying images. A copy of the written consent is available for review by the Editor-in-Chief of this journal.

## Competing interests

The authors declare that they have no competing interests.

## Authors’ contributions

NM performed the histological examination and diagnosis of the colonic polyp. JB and LR conducted a thorough literature review on osseous metaplasia in the gastrointestinal tract and were the major contributors in writing the manuscript. All authors read and approved the final manuscript.
